# Drosophila homologue of Diaphanous 1 (DIAPH1) controls the metastatic potential of colon cancer cells by regulating microtubule-dependent adhesion

**DOI:** 10.18632/oncotarget.4094

**Published:** 2015-05-25

**Authors:** Yuan-Na Lin, Ridhirama Bhuwania, Kira Gromova, Antonio Virgilio Failla, Tobias Lange, Kristoffer Riecken, Stefan Linder, Matthias Kneussel, Jakob R. Izbicki, Sabine Windhorst

**Affiliations:** ^1^ Department of General, Visceral and Thoracic Surgery, University Medical Center Hamburg-Eppendorf, Hamburg, Germany; ^2^ Department of Biochemistry and Signal Transduction, University Medical Center Hamburg-Eppendorf, Hamburg, Germany; ^3^ Institute for Microbiology, Virology and Hygiene, University Medical Center Hamburg-Eppendorf, Hamburg, Germany; ^4^ Department of Molecular Neurogenetics, Center for Molecular Neurobiology, ZMNH, University Medical Center Hamburg-Eppendorf, Hamburg, Germany; ^5^ Microscopy Imaging Facility, University Medical Center Hamburg-Eppendorf, Hamburg, Germany; ^6^ Department of Anatomy and Experimental Morphology, University Medical Center Hamburg-Eppendorf, Hamburg, Germany; ^7^ Department of Stem Cell Transplantation, University Medical Center Hamburg-Eppendorf, Hamburg, Germany

**Keywords:** colon cancer, cytoskeleton, formins, cellular adhesion, metastasis

## Abstract

Drosophila homologue of Diaphanous 1 (DIAPH1) regulates actin polymerization and microtubule (MT) stabilization upon stimulation with lysophosphatidic acid (LPA). Recently, we showed strongly reduced lung metastasis of DIAPH1-depleted colon cancer cells but we found accumulations of DIAPH1-depleted cells in bone marrow. Here, we analyzed possible organ- or tissue-specific metastasis of DIAPH1-depleted HCT-116 cells. Our data confirmed that depletion of DIAPH1 strongly inhibited lung metastasis and revealed that, in contrast to control cells, DIAPH1-depleted cells did not form metastases in further organs. Detailed mechanistic analysis on cells that were not stimulated with LPA to activate the cytoskeleton-modulating activity of DIAPH1, revealed that even under basal conditions DIAPH1 was essential for cellular adhesion to collagen. In non-stimulated cells DIAPH1 did not control actin dynamics but, interestingly, was essential for stabilization of microtubules (MTs). Additionally, DIAPH1 controlled directed vesicle trafficking and with this, local clustering of the adhesion protein integrin-β1 at the plasma membrane. Therefore, we conclude that under non-stimulating conditions DIAPH1 controls cellular adhesion by stabilizing MTs required for local clustering of integrin-β1 at the plasma membrane. Thus, blockade of DIAPH1-tubulin interaction may be a promising approach to inhibit one of the earliest steps in the metastatic cascade of colon cancer.

## INTRODUCTION

In mammalian cells three different isoforms of DIAPH (Drosophila homologue of Diaphanous; DIAPH1–3) have been identified [1–3]. DIAPH belongs to a group of actin regulatory proteins, which are defined by the presence of formin homology 1 (FH1) and formin homology 2 (FH2) domains, with intrinsic and conserved functions regulating cytoskeletal dynamics. In particular, the DIAPH isoform 1 (DIAPH1) has become subject of intense research. Over the past few years, DIAPH1 has been recognized as a key molecule controlling a large variety of cellular and morphogenetic functions in physiological, as well as pathological settings [4, 5]. Under physiological conditions DIAPH1 is mainly expressed in immune cells [6], but up-regulated expression has also been found in many cancer diseases [5, 7–11].

Functionally, DIAPH1 stimulates linear actin polymerization by binding F-actin by its FH2 and profilin/G-actin by its FH1 domains. The FH2 domains dimerize in order to associate with and cap the barbed end of an actin filament, preventing binding of capping proteins [12]. The FH1-domains recruit G-actin to the growing barbed end, resulting in polarized linear elongation of an actin filament. However, when cells are not specifically stimulated with lysophosphatidic acid (LPA), the auto inhibitory interaction of DID (Diaphanous inhibitory domain) and DAD (Diaphanous auto inhibitory domain) domain attenuate binding of actin to the FH1/FH2 domains. After binding of LPA to its G-protein coupled receptor, RhoA is activated and binds to the GTPase binding domain (GBD), the association of the inhibitory domains with GBD disrupts, thus releasing the FH1 and FH2 domains for binding with actin [13, 14]. Besides directly modulating the cytoskeleton, activated DIAPH1 has also transcriptional consequences by regulating the Serum Response Factor (SRF). For full activation, SRF must be associated with its co-activator myocardin-related transcription factor-A (MRTF-A), which is sequestered in the cytoplasm via interaction with G-actin. DIAPH1-mediated decrease of cellular G-actin causes the co-activator to translocate from the cytoplasm into the nucleus, where it activates SRF-dependent transcription. Thus, DIAPH1-mediated decrease of cellular G-actin concentration leads to the induction and up-regulation of SRF target genes, of which many appears to be essential for cell adhesion and spreading [15, 16].

In addition to its actin-modulating activity, DIAPH1 binds to and regulates the selective stabilization of microtubules (MTs) via its FH2 domains [17]. *In vitro*, DIAPH1 alone is sufficient to stabilize MTs, *in vivo* it functions as a scaffold protein with the tumor suppressor Adenomatous Polyposis Coli (APC) and Endbinding protein 1 (EB1), stabilizing MTs as a complex [18, 19]. Based on these different properties, DIAPH1 regulates many actin and tubulin-driven cellular effects: It is essential for formation of filopodia and invadopodia, for vesicle trafficking and for spindle formation [5]. In immune-cells these activities are required for cell motility during defense of infection and also tumor cells with ectopic expression of DIAPH1 show increased cell motility and invasion [4]. However, the relationship between its regulatory role in both, actin polymerization and MT stabilization, still remains elusive. Recently, we found DIAPH1 being specifically up-regulated in patient samples from colorectal carcinomas and found a positive correlation between DIAPH1 expression and the presence of colon cancer metastasis. In addition, we demonstrated that down-regulation of DIAPH1 in the three coloncarcinoma cell lines lines HCT-116, HT-29 and HROC-24, significantly decreased *in vitro* adhesion, invasion and migration. This insight of its metastasis-promoting activity in colon cancer cells was additionally confirmed by a subcutaneous SCID mouse model, showing that lung metastasis of HCT-116 cells was almost completely blocked after depletion of DIAPH1. However, since we have detected an accumulation of DIAPH1-depleted cells in bone marrow aspirates of SCID mice, we could not exclude that DIAPH1 depletion promotes metastatic outgrowth in organs other than lung [20].

Different from other studies showing DIAPH1-mediated cytoskeletal effects upon lysophosphatidic acid (LPA) stimulation [18, 19], our previous studies were all based on non-stimulated cells [20]. LPA mainly accumulates at sites of wound healing, where it is required for platelet activation and for stimulation of endothelial stress fiber formation [21]. Moreover, LPA recruits tumor cells to the sites of wound healing where tumor cell invasion into the adjacent tissue is facilitated [22] and LPA increases vascular permeability of endothelial cells during tumor cell extravasation [23]. According to our previous data, DIAPH1 showed to be essential for colon cancer metastasis, though not specifically stimulated with LPA.^19^ Therefore, we have outlined two objectives in this study: 1. we aimed to determine whether DIAPH1-depleted human colon cancer cells show organ- or tissue-specific metastases besides the lungs. 2. We aimed to analyze DIAPH1-effects on cellular adhesion and cytoskeletal dynamics in colon cancer cells that were not specifically stimulated with LPA.

## RESULTS

### DIAPH1 is essential for metastasis of colon cancer cells in SCID mice

Recently we have revealed that depletion of DIAPH1 in colon cancer cells (HCT-116 cells) strongly reduced lung metastasis in SCID mice [20]. However, we also found that the number of disseminated tumor cells (DTCs) in bone marrow was 4-fold higher in DIAHP1-depleted cells compared to the control. Thus, we could not exclude that DIAPH1 depletion promotes the formation of metastases in the bone marrow or other distant organs outside the lungs. Based on this consideration, here we analyzed HCT-116 control and DIAHP1-depleted HCT-116 cells (termed as D5 cells, see [20]) cells stably expressing luciferase regarding their dissemination in SCID mice by bioluminescence imaging (BLI). At injection, D5 cells exhibited an about 60% down-regulation of DIAPH1 (Figure S1).

**Figure 1:**
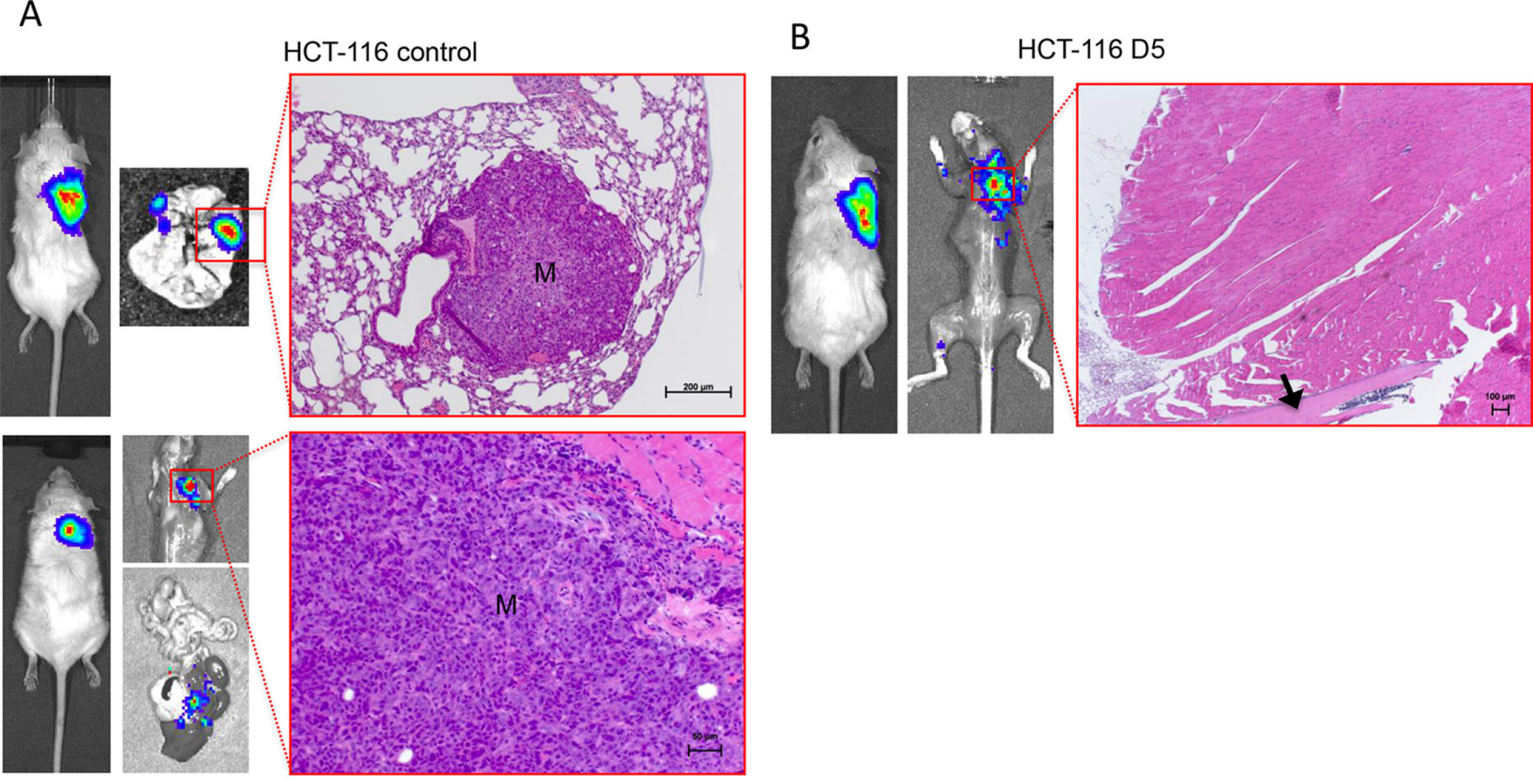
Bioluminescence imaging (BLI) of spontaneous metastasis formation after surgical resection of primary tumors SCID mice injected with luciferase-over-expressing HCT-116 scrambled control and DIAPH1-depleted (D5) cells developed subcutaneous primary tumors of about 1.5 cm^3^ within 42 days in 100% and 71% of mice, respectively. Primary tumors were surgically resected and metastasis formation was monitored for another 40 to 44 days after surgery by regularly performed BLI. **A.** After this period, 100% of control mice revealed strong BLI signals mostly in the lungs (upper panel) and in the livers (lower panel). Histological examinations of H.E.-stained lung sections verified the presence of large pulmonary metastases (M) **B.** In the D5 group, spontaneous bone marrow metastases as expected from our previous study could not be detected after this period of postoperative monitoring. In contrast, only one mouse of the D5 group showed a rather diffuse BLI signal above the right scapula, which could not be verified by histology. Corresponding H.E.-stained serial sections showed large, tumor cell-free muscular tissues and an adjacent bone, presumably scapula (arrow). The corresponding BLI signal was therefore most likely due to relapsing subcutaneous primary tumor cells.

We found that all mice injected with control cells developed subcutaneous primary tumors, which were dissected after a mean period of 42 days after injection. After surgery, metastasis development could be monitored by BLI for another 40 days. After this period, BLI analysis revealed strong signals at different distant locations such as the lungs, livers, or soft tissues adjacent to bones. Histological examinations of these samples verified the presence of large lung metastases and tumor cell deposits surrounded by murine skeletal muscles, respectively, in the control group (Figure 1, left panel; metastasis are marked with “M”). Lung metastases were macroscopically visible and Alu-PCR analysis revealed an accumulation of an average of one million colon cancer cells in the lungs (Table 1).

**Table 1:** Effect of DIAPH1 depletion on dissemination of HCT-116 cells

	OP days	Necroscopy days	BLI positive mice %	Lung Cell No.	Bone marrow Cell No.	Blood Cell No.
Control	42	82	100	1058166	48	26
D5	42	86	25	581	281	66

Control and DIAPH1-depleted cells (D5) were subcutaneously injected into the neck of SCID mice and after 42 days the tumors were resected. Dissemination of tumor cells was monitored by BLI and 40 to 44 days after resection of tumors, mice were sacrificed and the number of tumor cells (Cell No.) in lung, bone marrow and blood was analyzed by Alu PCR. Shown are mean values of three to four different mice, injected with control or D5 cells, respectively.

In contrast, five of the seven mice injected with D5 cells formed tumors and were monitored by BLI for up to 44 days after dissection of the primary tumors. In this group, only one mouse showed a luciferase signal located in the soft tissue of the neck region at the end of the experiment. Histological examinations of corresponding sections did not reveal any metastatic cells (Figure 1B, right panel) so that the BLI signal was most likely due to recurrent primary tumor cells in this area. Alu-PCR confirmed that the number of DTc in the lungs of mice that received D5 cells was drastically reduced (by 99.95%; Table 1). In accordance with our previous data, the number of D5 cells in the bone marrow was 6-fold higher than that of control cells (Table 1). However, as we did not find any BLI signals in the skeleton of D5 mice after surgery, the accumulation of DIAPH1-depleted cells in the bone marrow as indicated by Alu-PCR was obviously not accompanied by an increased outgrowth of clinically detectable bone marrow metastases.

Summarized, these results confirm that depletion of DIAPH1 strongly reduces lung metastasis of HCT-116 cells and reveal that DIAPH1 knock down cells are not able to form metastasis outside the lungs.

### Dependence on adhesion of HCT-116 cells on DIAPH1 expression and on cytoskeletal dynamics

Since our previous data, showing significant effects of DIAPH1 on the metastatic potential of colon cancer metastasis *in vitro* and *in vivo*, were not based on LPA stimulation, one main goal of this study was to analyze DIAPH1-mediated cellular effects on non-stimulated HCT-116 colon cancer cells more closely. Since adhesion to the substrate is one of the earliest steps of tumor cell metastasis, we analyzed the effect of DIAPH1-depletion on early cell adhesion on collagen (two hours after seeding) of non-stimulated HCT-116 cells.

**Figure 2:**
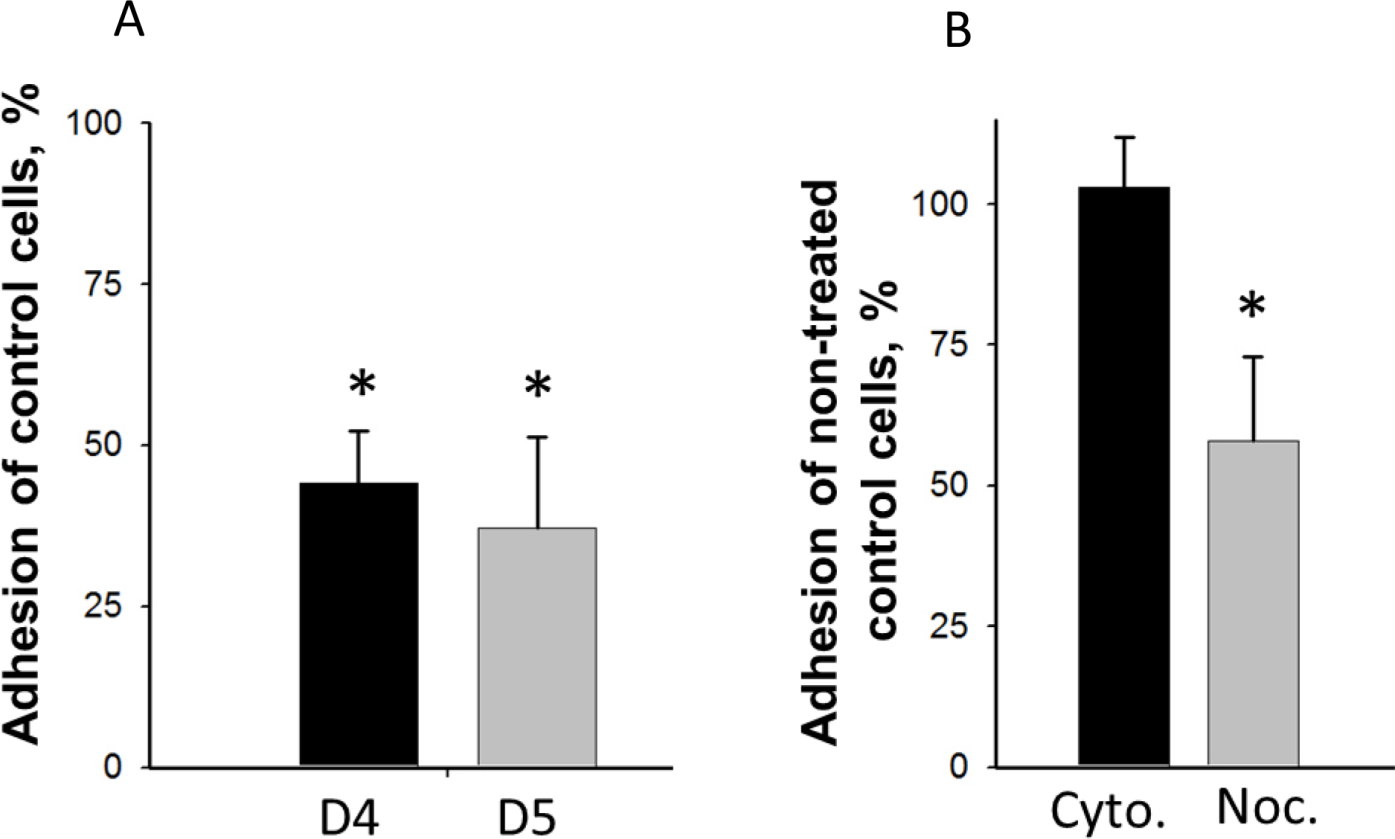
Effect of DIAPH1-depletion and inhibition of cytoskeletal dynamics on adhesion of non-stimulated HCT-116 cells **A.** 2 h after seeding of control and DIAPH1 knock down cells were washed with PBS, fixed and stained with DAPI. Cells were counted at Keyence BZ-9000 with the Hybrid Cell Count function. Mean values ± SD as percent of control of four independent experiments are shown. **B.** Scrambled control cells were treated with DMSO (control), 1 μM cytochalasin B for 1 h or with 100 μM nocodazole for 3 h, respectively. After the respective incubation times the cells were trypsinized, seeded in Petri dishes, incubated for 2 h and treated as described in **A.** Mean values ± SD as percent of control or as percent of non-treated control cells of four independent experiments are shown. D4 and D5: DIAPH1 knock down cells; Cyto: cytochalasin B-treated scrambled cells; Noc: nocodazole-treated scrambled cells, *p < 0.05.

We found, indeed, that even under non-stimulating conditions, depletion of DIAPH1 reduced adhesion of HCT-116 colon cancer cells to collagen by 50% (Figure 2A). As in absence of LPA-stimulation the actin-nucleating activity of DIAPH1 is blocked [19], it was unlikely that the formin controls cellular adhesion by its actin-nucleating activity. To analyze this assumption, actin and also tubulin dynamics were blocked by treating scrambled HCT-116 control cells with cytochalasin B or with nocodazole. Thereafter, early adhesion was compared between non-treated and treated cells. Trypan blue analysis showed that these treatments did not affect viability of HCT-116 cells (data not shown). Our data show that treatment with cytochalasin B did not affect adhesion while treatment with nocodazole significantly inhibited adhesion of HCT-116 scrambled cells by 42 ± 11% (Figure 2B). This result confirms our assumption that under non-stimulating conditions DIAPH1 does not control adhesion by it actin-nucleating activity. Since on the other hand adhesion was blocked in response to destabilization of MTs, it is very likely that the MT-stabilizing effect of DIAPH1 accounts for DIAPH1-controlled cellular adhesion.

### DIAPH1 is associated with both actin and MTs in HCT-116 cells under non-stimulating conditions

Our assumption that DIAPH1 controls adhesion of non-stimulated HCT-116 colon cancer cells by stabilizing MTs had to be proven because up to now the MT-stabilizing effect of DIAPH1 was only shown after preventing its auto-inhibition [18, 19]. Thus, we next analyzed the effect of DIAPH1 on cytoskeletal dynamics in non-stimulated HCT-116 cells.

**Figure 3:**
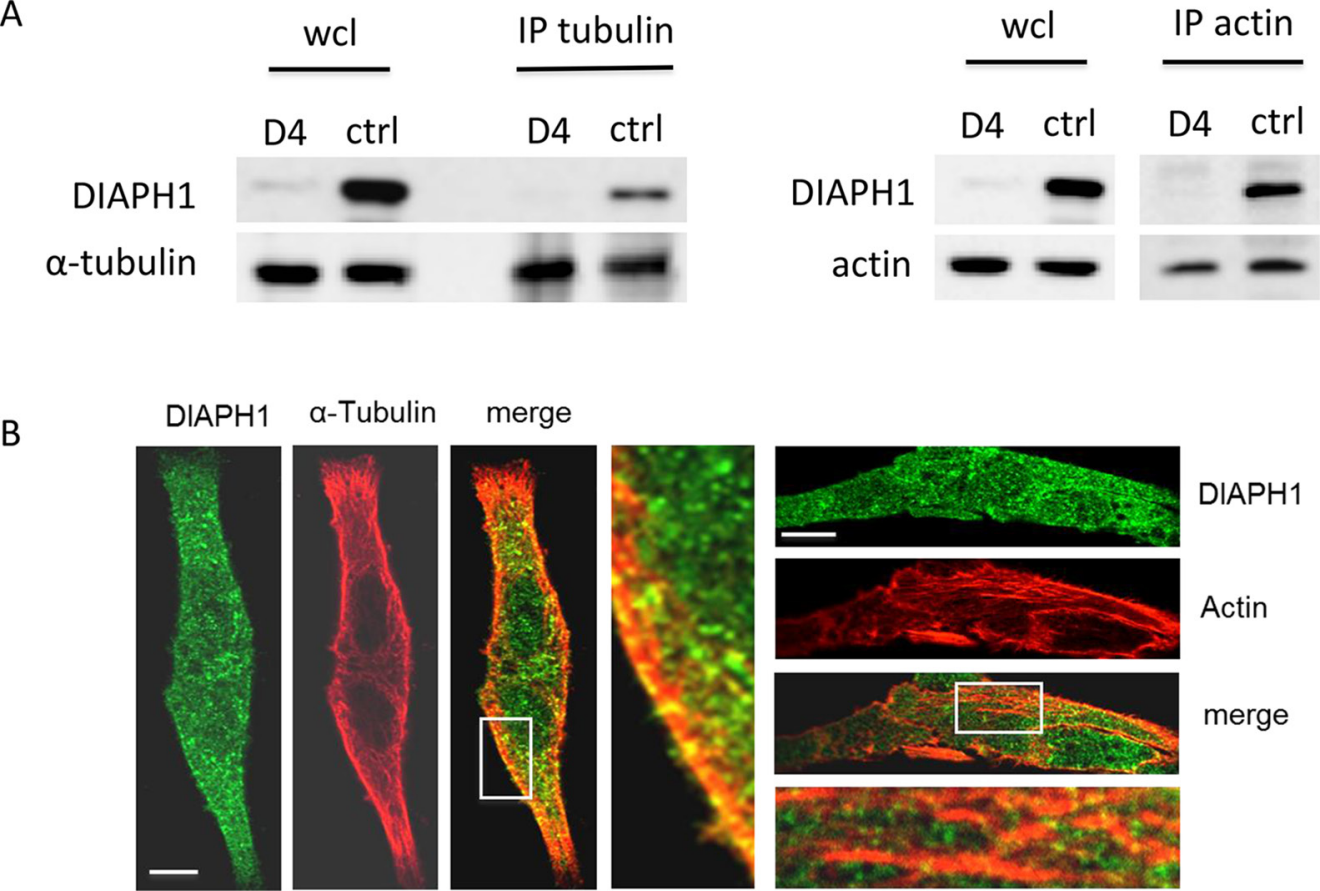
DIAPH1 co-localizes with actin and α-tubulin in non-stimulated HCT-116 cells **A.** Tubulin (left panel) or actin (right panel) was immunoprecipitated from scrambled control or DIAPH1 knock down cells, respectively. Binding of DIAPH1 to tubulin or to actin, respectively, was analyzed by Western blotting. **B.** Immunostainings of DIAPH1, α-tubulin and actin confirm co-localization (merge) of DIAPH1 with both, actin and α-tubulin in non-stimulated HCT-116 scrambled cells. Observation of DIAPH1 interaction with actin and α-tubulin was performed using confocal microscope TCS SP5 (Leica). D4: DIAPH1 knock down, ctrl: control: scrambled; wcl: whole cell lysate.

First, we analyzed the interaction of DIAPH1 with microtubules or with actin in non-stimulated HCT-116 cells. For this purpose, tubulin and actin were immunoprecipitated from HCT-116 control cell (ctrl) lysates and binding of DIAPH1 to tubulin and actin was analyzed by Western blotting. In addition, immunoprecipitates from lysates of HCT-116 DIAPH1 knock down cells (D4) were used as negative control. As shown in Figure 3A, DIAPH1 was clearly detectable in tubulin and actin-coupled beads incubated with cell lysates from control cells, whereas no signal was detected when the beads where incubated with cell lysates from DIAPH1 knock down cells. Thus, also in non-stimulated HCT-116 cells DIAPH1 seems to interact with both cytoskeletal elements. To verify this result, co-localization studies between DIAPH1 with tubulin or actin in HCT-116 control cells were performed. This evaluation revealed that DIAPH1 is homogeneously distributed all over the cell and partly co-localizes with tubulin and with actin (Figure 3B).

From these data we conclude that, although HCT-116 cells were not specifically stimulated, DIAPH1 partially interacts with both, tubulin and actin in HCT-116 cells.

### DIAPH1 controls microtubule but not actin dynamics in non-stimulated HCT-116 cells

To show if the partial localization of DIAPH1 to actin and to tubulin in non-stimulated HCT-116 cells has an impact on cytoskeletal dynamics, we analyzed the G- to F-actin ratio, the fluorescence-signals of phalloidin-stained actin filaments and tubulin dynamics in control and DIAPH1-depleted cells. The G- to F-actin ratio was analyzed by the well-established DNAse-assay [24] and tubulin dynamics by quantification of detyrosinated (thus stabilized) microtubules.

**Figure 4:**
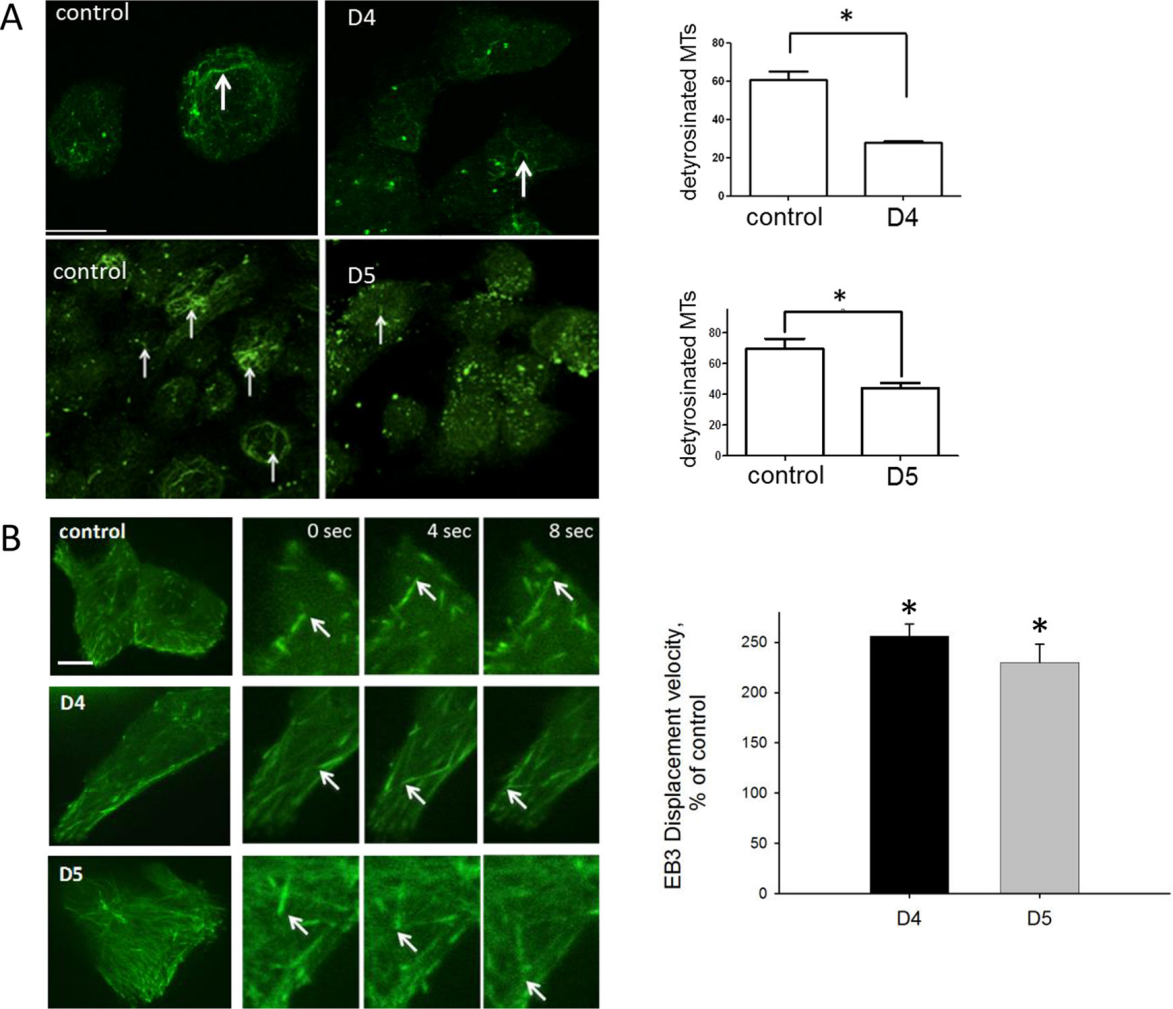
DIAPH1 stabilizes microtubules and reduces microtubule dynamics **A.** Cells were seeded on coverslips for 2 h, fixed with methanol and stained for detyrosinated microtubules (green). Images are extended projections of Z-stacks. White arrows indicate detyrosinated (thus stable) microtubules. Right panel: Cells with detyrosinated MTs and those without were quantified using the software Volocity 6.0. Shown are the percentages of cells containing detyrosinated MTs, respectively. Mean values ± SD of three independent experiments are shown. **B.** Cells were seeded on coverslips and transfected with EB3-eGFP. Time-lapse experiments were performed at Spinning Disk (Yokogawa, 100x magnifications). Pictures were taken in an interval of 2 s. The whole time interval for each video clip was 5 min, at least 10 videos were performed for each cell line. Shown are representative pictures of EB3-eGFP transfected cells for scrambled and both DIAPH1 knock down cell lines, D4 and D5, arrows indicate EB3 signals. Right panel: Analysis was performed using the software Volocity 6.0. with time-dependent movement of EB3 signals at MT. Shown are the EB3 displacement velocities of both DIAPH1 knock down cell lines, D4 and D5, as percent of control. Mean values ± SD of three independent experiments are shown. D4 and D5: DIAPH1 knock down cells, control: scrambled; MTs: microtubules; Bar, 10 μm, *p < 0.05.

We found no significant differences in the G- to F-actin ratio between control and DIAPH1-depleted cells (Table 2) and also fluorescence-signals of phalloidin-stained actin filaments were not significantly different between these cell lines (Figure S2). However, a significant reduction of detyrosinated MTs in DIAPH1 knock down cells was observed (Figure 4A). In order to exclude this observation being due to general MT disintegration in DIAPH1-depleted cells, we further analyzed MT dynamics of the cells. For that purpose the velocity of growing microtubule plus tips was measured. Cells were transfected with EGFP-tagged end binding protein 3 (EB3), which labels MT growing plus ends [25]. Live cell imaging of EB3-labeled microtubules revealed that velocity of MT growing ends was doubled in DIAPH1 knock down cells (Figure 4B). Thus, although in non-stimulated HCT-116 cells DIAPH1 partially localizes to actin and to tubulin, its depletion only significantly affects the modulation of microtubules.

**Table 2:** Impact of DIAPH1 on F-actin/G-actin ratio

	G-Actin, %	F-Actin, %
Control	53.5 ± 4.4	46.5 ± 4.4
DIAPH1 kd	51.4 ± 4.5	48.6 ± 4.5

The F-actin/G-actin ratio was examined in non-stimulated HCT-116 scrambled control and HCT-116 DIAPH1 knock down (kd) D5 cells by using the DNAase-Assay^20^. Mean values ± SD of five different experiments are shown.

### DIAPH1 controls MT-dependent cell adhesion processes in HCT-116 cells

Our data that under non-stimulating conditions DIAPH1 is required to stabilize MTs together with the finding that adhesion of HCT-116 cells depends on tubulin dynamics, further supports our presumption that DIAPH1 controls adhesion of HCT-116 cells by its MT-stabilizing activity. As a further verification that DIAPH1 controls adhesion by stabilizing MTs, we examined if DIAPH1 is essential for MT-dependent adhesion processes. For this purpose, we compared cell spreading, clustering of the main focal adhesion protein integrin-β1 at focal adhesion like structures (FA) [26], and vesicle trafficking of control and DIAPH1-depleted cells.

**Figure 5:**
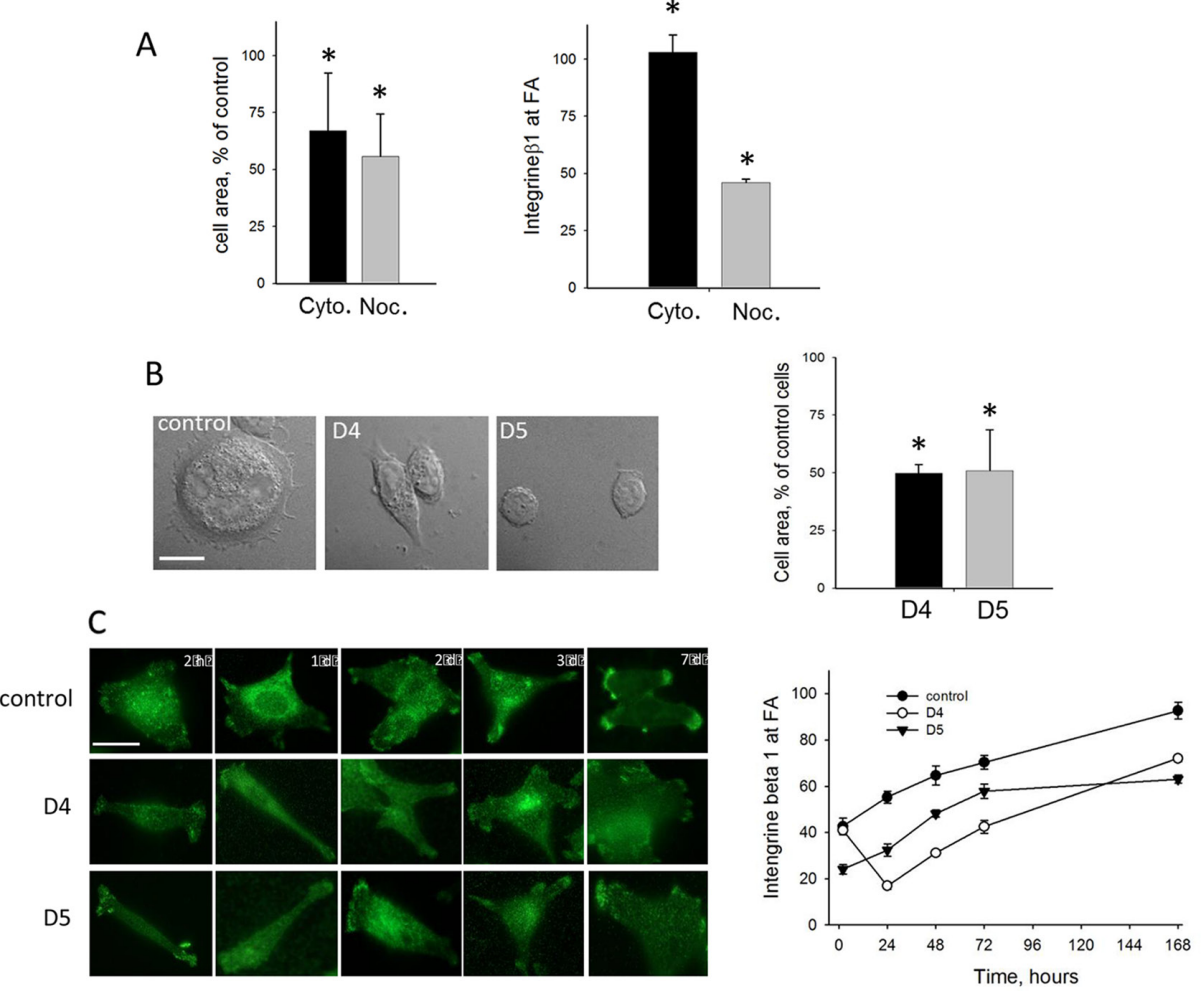
Effects of DIAPH1-depletion in HCT-116 cells on cytoskeletal dynamics, cell spreading and formation of focal adhesions Fas **A.** Scrambled control cells were treated with DMSO (control), 1 μM cytochalasin B for 1 h or with 100 μM nocodazole for 3 h, respectively. After the respective incubation times the cells were trypsinized, seeded in Petri dishes, incubated for 2 h and area (left panel) or translocation of integrin-β1 to focal adhesions (right panel) of fixed cells was analyzed. Cell area was analyzed by using the Area Measure function of Keyence BZ-9000 microscope. Translocation of integrin-β1 to focal adhesions was analyzed by staining the cells for integrin-β1 and analyzing them by a Zeiss Apotome fluorescent microscope. Shown are mean ± SD as percent of scrambled control cells of at least 100 cells. **B.** Cell area of non-treated control and DIAPH1 knock down cells (D4 and D5) cells was analyzed as described above. **C.** 2 h, 24 h, 48 h, 72 h or 168 h after seeding, cells were fixed and stained for integrin-β1. Analysis was performed at Zeiss Apotome fluorescent microscope. Left panels show representative micrographs and right panels mean values ± SD of cells with distinct integrin-β1 signals at FA, given as percentage of total evaluated cells. For each cell line and at each time point at least 80 cells were analyzed. Bar, 10 μm, *p < 0.001.

First, we analyzed if spreading and clustering of integrin-β1 at FAs is only dependent on tubulin or also on actin dynamics by nocodazole- or cytochalasin B-treatment of control HCT-116 cells, respectively. Our data showed that spreading was dependent on both, tubulin and actin dynamics, while clustering of integrin-β1 at adhesion sites was only dependent on MT dynamics (Figure 5A). On the other hand, depletion of DIAPH1 reduced spreading as well as translocation of integrin-β1 to FA (Figure 5B, 5C).

To analyze the impact of DIAPH1 on vesicle trafficking, BDNF-vesicle trafficking was analyzed and served as representative for general vesicle trafficking in HCT-116 cells. Four measurement categories were defined beforehand: 1. number of bidirectionally moving vesicles, 2. vesicle accumulations per cell (defined by at least 3 closely located vesicles), 3. number of linearly moving vesicles per cell and 4. track length of vesicles with linear movement. The data revealed that the percentage of bidirectionally moving vesicles per cell and the percentage of vesicle accumulations per cell were both increased about 2-fold in DIAPH1 knock down cells. Instead, the percentage of cells with linearly moving vesicles was decreased 9-fold and the average track length of linearly moving vesicles (shown as % of whole cell length) was decreased 2-fold in DIAPH1 knock down cells (Table 3). To show if BDNF-trafficking is only dependent on microtubules or also on actin dynamics, scrambled control cells were treated with nocodazole or cytochalasin B. Nocodazole-treated scrambled cells showed comparable vesicle trafficking phenotype to DIAPH1 knock down cells, whereas cytochalasin B treatment caused no effect on BDNF-trafficking and was similar to that of DMSO control cells (Table 3). In conclusion, these data show that in non-stimulated HCT-116 cells directed vesicle trafficking is only dependent on tubulin but not on actin dynamics and reveal that depletion of DIAPH1 exhibits the same effect on vesicle trafficking as destabilization of MTs.

**Table 3:** DIAPH1 controls tubulin-dependent vesicle trafficking

	pendling vesicles (%)	vesicle accumulation (%)	vesicles with linear movement (%)	track length of linear moving vesicles (% of cell length)
Control	18.7 ± 9.2	6.3 ± 3.2	64.6 ± 11.3	29.7 ± 4
D4	42 ± 18^*^	16.4 ± 4.9^*^	6.5 ± 7.7^*^	12.9 ± 0.9^*^
D5	51.2 ± 11.3^*^	12.7 ± 3.3^*^	7.8 ± 6.5^*^	14.2 ± 3.6^*^
Nocod.	50.3 ± 16.7^*^	14.5 ± 5.3^*^	5.0 ± 3.6^*^	11.6 ± 3.2^*^
Cyto.	18.5 ± 5.5	6.9 ± 3.0	75.9 ± 6.3	29.6 ± 4.9

Control and DIAPH1 depleted cells (D4, D5) were transfected with EGFP-BDNF to visualize vesicle trafficking. In addition, EGFP-BDNF expressing control cells were treated with 100 μM Nocodazole for 3 hours and with 1 μM Cytochalasin B for 1 hour. Mean values ± SD of three independent experiments are shown.

* *p* > 0.05. Nocod; Nocodazole; Cyto; cytochalasin B.

In summary, these data demonstrate that vesicle trafficking and clustering of integrin-β1 at FA were only dependent on tubulin while cell spreading was dependent on both, actin and tubulin dynamics. Overall, our data show that adhesion of non-stimulated HCT-116 cells is dependent on DIAPH1-mediated MT-stabilization. Furthermore, we show that DIAPH1 is essential to stabilize MTs, which are required for directed vesicle trafficking and translocation of integrin-β1 to FAs.

Based on these results, we conclude that non-stimulated DIAPH1 is sufficient to control translocation of the main focal adhesion protein integrin-β1 to the cell surface, which explains impaired cell adhesion and thus reduced metastasis of DIAPH1-depleted HCT-116 cells.

## DISCUSSION

Colon cancer is a leading cause of morbidity and mortality worldwide and formation of distant metastasis is prognostically the most important moment in the development of malign epithelial tumors [27, 28]. Metastasis is a complex process, going along with strong morphological and functional changes of tumor cells. One of the earliest steps of metastasis is the adherence of tumor cells to the extracellular matrix (ECM) or the vascular endothelium, in order to escape from the primary tumor to invade surrounding blood vessels, or to extravasate from blood vessels to set distant metastasis [29].

In this study, we confirmed our recent finding [20] that reduction of DIAPH1 expression strongly inhibited lung metastasis of HCT-116 cells in a subcutaneous SCID mouse model. Our data showed that knock down of DIAPH1 expression reduced lung metastasis by 99.95%, whereby lung metastasis was only detected in one out of six mice injected with DIAPH1-depleted cells. In addition, we confirmed accumulation of DIAPH1 knock down cells in bone marrow, which led us to assume that DIAPH1-depleted cells might form bone metastasis. However, in mice injected with DIAPH1 knock down cells, no metastasis outside the lungs were detected, indicating that the cells accumulated in blood vessels of bone marrow. The finding, that DIAPH1-depleted cells are not able to leave the blood vessels, shows that, in addition to invasion, also extravasation is impaired in DIAPH1 knock down HCT-116 cells.

Mechanistically, the strong effect of DIAPH1 depletion on metastasis of colon cancer cells can be explained by our result that DIAPH1 is essential for one of the earliest steps in the metastatic cascade; cellular adhesion to the ECM. Interestingly, our data showed that this process was controlled by DIAPH1 even without LPA-stimulation. This result was surprising because in absence of LPA-mediated stimulation of RhoA, DIAPH1 is in its auto-inhibited form, preventing FH1 and FH2 interplay required for actin nucleation [18, 19]. However, our data showed that DIAPH1 partially interacts with both, tubulin and actin in HCT-116 cells but we revealed that depletion of DIAPH1 did not affect actin dynamics of HCT-116 cells in absence of LPA stimulation which confirmed that DIAPH1 did not significantly stimulate the production of F-actin under these conditions. This observation, together with our finding that inhibition of actin dynamics by cytochalasin B did not affect adhesion of non-stimulated HCT-116 colon cancer cells, strongly indicate that DIAPH1-controlled cellular adhesion was not mediated by the actin-nucleating activity of DIAPH1. On the other hand, we found that knock down of DIAPH1-expression significantly impaired MT-stabilization in non-stimulated HCT-116 cells, indicating that FH2-mediated stabilization of MTs also works in the auto-inhibited state of DIAPH1. Additionally, we show that in non-stimulating HCT-116 cells vesicle trafficking and translocation of integrin-β1 to FAs were dependent on DIAPH1 expression and on stable MTs but not on actin dynamics. As integrin-β1 directly binds ECM proteins, it mediated anchorage of tumor cells to the substrate and thus is essential to initiate and to stabilize cellular adhesion [26, 30, 31]. Accordingly, decreased clustering of integrin-β1 at the plasma membrane impairs cellular adhesion. Based on these considerations, we conclude that reduced adhesion of non-stimulated DIAPH1-depleted HCT-116 cells at least in part results from impaired focal clustering of integrin-β1. In summary our data demonstrate that also in absence of LPA stimulation, DIAPH1 is essential for MT-dependent cellular adhesion of HCT-116 colon cancer cells, thus facilitates one of the first steps in the metastatic cascade. LPA-stimulation may enhance this effect by additionally activating the actin nucleating activity of DIAPH1 to stimulate formation of cellular protrusion required for tumor cell invasion [29].

To our knowledge this is the first study showing that even under basal conditions DIAPH1 is required for stable MT-dependent metastasis of colon cancer cells. Accordingly, we suggest a direct interaction between the FH2 domain of DIAPH1 and tubulin, even being in its auto-inhibited state. However, it leaves future studies to reveal whether this assumption proves true on a molecular level, or whether other factors besides LPA also play a regulative role in DIAPH1-mediated MT-stabilization in non-stimulated colon cancer cells. Since the FH2 domain is essential for all known DIAPH1-mediated cellular effects, identification of drugs specifically blocking the interaction of FH2 with tubulin and actin, respectively, may be a promising new therapeutic approach for metastasizing colon cancers.

## EXPERIMENTAL PROCEDERS

### Cell line

HCT-116 cells were obtained from the *Deutsche Sammlung von Mikroorganismen und Zellkulturen* (Braunschweig, Germany). The cell line HCT-116 was cultured in Dulbecco's modified Eagle's medium (DMEM, Gibco), supplemented with 10% (v/v) fetal calf serum (FCS), 100 μg/ml streptomycin, and 100 units/ml penicillin. Cells were cultured at 37°C in a humidified incubator with 5% CO_2_. The DIAPH1 knock down D4 and D5, and scrambled cell lines were additionally treated with 5 mg/ml puromycin for selection.

### Stable knock down of DIAPH1

Stable knock down of DIAPH1 expression in HCT-116 cells was performed as described [20]. Five different vectors (pLKO.1 shRNA) from Sigma, coding for shRNA against DIAPH1 were tested [20]. With two of them (shRNA 4 and shRNA 5) a 60% (shRNA 5) to 95% (shRNA 4) reduced expression was achieved (Supplementary Figure 1). Cells stably expressing scrambled shRNA served as control. All *in vitro* data shown here were reproduced with both DIAPH1 shRNAs and the corresponding cell line was termed D4 (DIAPH1 shRNA 4 knock down) and D5 (DIAPH1 shRNA 5 knock down). The shRNA vector (pLKO.1) has a puromycin resistance, therefore knock down cells and scrambled were selected with puromycin (5 mg/ml).

### Generation of viral particles and luciferase transduction of HCT-116 cells

Viral particles were produced as cell-free supernatants by transient transfection of the packaging cell line HEK-293T as described [32]. In brief, the lentiviral vector LeGO-iG2-Luc2 expressing Luc2-cDNA (Firefly luciferase, Promega) was packaged using the third-generation packaging plasmids pMDLg/pRRE (http://Addgene.org #12251), pRSV-Rev (http://Addgene.org #12253) and phCMV-VSV-G expressing the envelope protein of vesicular stomatitis virus. The supernatant was harvested 24 hours after transfection, 0.45 μm filtrated and stored at −80°C. Titration on 293T cells using 8 μg/ml polybren and spin-inoculation (1000 × g, 1 h, 25°C) resulted in a functional titer of 5.2 × 10^7^ TU per ml. HCT-116 target cells were plated at 5 × 10^4^ cells in 0.5 ml medium in each well of a 24 well plate. After addition of 30 μl of viral particle containing supernatant to the cells, the medium was replaced the next day.

### *In vivo* monitoring of spontaneous metastasis formation in a xenograft SCID mouse model

SCID mice were obtained from Charles River (Sulzfeld, Germany), maintained in a pathogen-free environment and all experiments were approved by the local animal experiment approval committee (Project No. G09/59). Luciferase (Luc2)- and eGFP-over-expressing HCT-116 control and D5 cells were xenotransplanted into 17-week-old SCID mice by subcutaneous injection of 1 × 10^6^ cells/200 μl cell culture medium per mouse (*n* = 6 and *n* = 7 in the control and D5 group, respectively). Female and male mice were equally distributed. As soon as primary tumors reached about 1.5 cm^3^, the tumors were surgically resected [33], weighed and processed for subsequent analyses. Due to inoperability of the primary tumor, one mouse of the scrambled group and one mouse of the D5 group had to be euthanized in accordance with the animal welfare requirements [34]. After surgery, metastasis formation was monitored by regular, whole-body bioluminescence imaging (BLI) scans (Core Facility Optical Imaging, UKE) following intraperitoneal application of 200 μl firefly luciferin (30 mg/ml from BioSynth). Once metastatic lesions were identified in any region of the body, the respective mice were subjected to terminal cardiocentesis and the organs/bones of interest were dissected immediately in order to re-evaluate the BLI-signals *ex vivo*. BLI-signals were detectable for up to 45 min *post injection* and hence about 40 min *post mortem*. Afterwards, BLI-positive organs were fixed in 4% formalin, embedded in paraffin, and processed for hematoxylin/eosin-staining on 5 μm thin sections. Affected bones were decalcified using 10% EDTA (in 0.1 M phosphate buffer, pH 7.4, 37°C, for approx. 48 h) [33] prior to paraffin embedding. Independent of the results from the *in vivo*-BLI scans, the left lungs and left hind limbs from all mice were excised and processed for biomolecular (DNA-based) quantification of circulating/disseminated tumor cells by *Alu*-PCR as described [20, 34]. As soon as all mice from the control group revealed metastatic lesions as detected by BLI, all mice from the D5 group were injected with luciferin, sacrificed and all organs and the bare skeleton were scanned to exclude even smaller metastatic lesions.

### DNase I inhibition assay for determination of intracellular actin

The DNAse I inhibition assay for measuring the G- to F-actin ratio in cells, was performed according to Katsantonis et al.[24]. The principle of this assay is that only G-actin and not F-actin inhibits the activity of DNAse I. The cellular G-actin content is directly determined from cell lysates by measuring inhibition of DNAse I. Total cellular actin was determined after treatment of cell lysates with guanidine HCl to depolymerize F-actin. The F-actin content was calculated by subtracting the G-actin amount from the total cellular actin (F-actin = total actin − G-actin). The experimental procedure was performed according to Windhorst et al. [35].

### Adhesion assay

For adhesion assay 1 × 10^5^ HCT-116 cells were seeded on 60 mm Tissue Culture Dishes (TPP) coated with collagen G. After 2 h cells were washed three times with PBS in a standardized way and fixed with 4% paraformaldehyde/PBS. Cells were stained with 4′, 6-diamidino-2-phenylindole (DAPI). The cell number was examined by counting the DAPI stained nuclei with the Hybrid Cell Count function at Keyence BZ-9000. Analyses were performed in duplicates and as independent experiments four times.

### Nocodazole and cytochalasin B treatment

Nocodazole (Tocris Bioscience) and cytochalasin B (Sigma-Aldrich) treatment was performed in scrambled control HCT-116 cells. Nocodazole was used in a concentration of 100 μM, cells were incubated for 3 h prior to experiments. Cytochalasin B was used in a concentration of 1 μM, cells were incubated for 1 h prior to measurements. In all cases DMSO treated cells served as controls. In order to prove significant effects with the used working concentrations on actin and tubulin, immunostainings and subsequent observation of the respective cytoskeleton structures by fluorescence microscopy were performed prior to experiments.

### Cell spreading assay

During cell spreading the former round shaped suspended cells flatten to the substrate and with this their cell areas increase. Therefore, the effect of spreading on drug treatment or depletion of DIAPH1 was examined by comparing cell areas of treated and non-treated cells. 0.5 ml of cell suspension (1 × 10^5^ cells/ml medium) were transferred into 35 mm cell culture dishes with glass bottom (MatTek) coated with collagen G (Biochrom). After 2 hours, cells were fixed with 4% paraformaldehyde/PBS. Quantification of cell area was performed using the Area Measure function at Keyence BZ-9000. Analyses were done in duplicates and as independent experiments at least three times.

### Immunoprecipitation

Binding of 50 μl anti-mouse-IgG-agarose to 5 μl anti-actin (sc17827, Santa Cruz) or anti-α-tubulin (T9026, Sigma-Aldrich) antibody was performed at 4°C for 2 h. Beads were washed 3-times with PBS, prior to adding the cell lysate and the sample was incubated at 4°C overnight. After washing the beads 5 times with MPER lysis buffer (Promega) SDS sample buffer was added to the agarose-beads, and the mixture was boiled at 95°C for 5 min. The beads were removed by centrifugation, and the supernatants were subjected to SDS-PAGE electrophoresis. Proteins were subsequently blotted onto nitrocellulose membranes, and binding of DIAPH1 was examined by Western blotting.

### Western blot

Western blot analysis was performed as described [35]. Protein expression was quantified using an LAS-3000 Imager from Fuji (Raytest, Straubenhardt, Germany).

### Immunostaining

For immunofluorescence analysis of DIAPH1 (ab11173, abcam), actin (Alexa 468 phalloidin, Life Technologies), α-tubulin (T9026, Sigma-Aldrich) and integrin-β1 (sc-73610, Santa Cruz), 2.5 × 10^4^ cells were seeded in 8-chamber slides (ibidi) coated with collagen IV, grown to 50% confluence, washed with PBS and fixed with 4% paraformaldehyde/PBS for 10 min at 37°C. After incubation for 5 min with Triton-X-100 at room temperature, fixed cells were blocked in 5% BSA/PBS for 20 min at room temperature. The primary antibodies were used in a dilution of 1:200 in PBS and incubated at 4°C over night. Secondary antibodies were used in a dilution of 1:1000 in PBS and incubation was done for 1 h at room temperature. After washing with PBS, stained cells were used for analysis with fluorescence microscopy. For each cell line and at each time point at least 80 cells were analyzed. Analyses were performed in duplicates and as independent experiments at least three times. For co-localization studies of DIAPH1 with actin and tubulin, the confocal microscope TCS SP5 (Leica) was used. To stain F-actin, fixed cells were incubated with Alexa 468 phalloidin diluted 1:500 in PBS at room temperature for 30 min. For immunofluorescence analysis of detyrosinated microtubules, 5 × 10^4^ cells were seeded on coverslips and incubated for 2 h. Cells were fixed with ice cold methanol at −20°C for 5 minutes. All washing steps were performed with 0.05% Triton X/PBS. Blocking was performed with 1% BSA in 0.05% Triton X/PBS for 1 h. Anti-detyrosinated α-tubulin antibody (ab48389, abcam) was diluted 1: 200 in blocking solution. Coverslips were incubated with primary antibody solution for 1 h. Secondary antibody, Anti-rabbit antibody conjugated to 488 (Jackson immunoresearch), was diluted 1:200 in 0.05% Triton X/PBS. Coverslips were mounted on slides using anti-fading agent Movial. Analysis was performed at TCS SP5 (Leica) and Volocity 6.0 (PerkinElmer) was used for further evaluation.

### Transient transfections

Transient transfections were performed using Lipofectamine reagent (Invitrogen), according to the manufacturer's instructions. Transfected cells were cultured for 48 h, fixed with 4%paraformaldehyde/PBS or used for Life Cell imaging. The vector EB3-eGFP was a gift from Anna Akhmanova [25]. The vector BDNF-eGFP was cloned from cDNA of mouse hippocampus and was inserted in GFP-N1 vector clontech with *Bam* HI, *Hind* III restriction enzymes.

### Time-lapse experiments

Cells were seeded on coverslips placed in 6-well-plates. After 48 h transient transfections were performed as described above, cells were incubated for 24 h before starting time-lapse experiments. The coverslips were taken out of the 6-well-plates and placed in a “coverslip cage”, protective of evaporation. During Live cell imaging, cells were kept at constant temperature (37°C) and CO_2_ concentration (5%). Images were acquired using Spinning Disk (Yokogawa) Live Cell Confocal (SDC) technology (Visitron Systems Puchheim, Germany), with solid state lasers: 488, 561, 647 or 405. SDC was combined with two charge-coupled device EM-CCD cameras (Hamamatsu Photonics 512/1024, Herrsching am Ammersee, Germany) and equipped with an optical image splitter for simultaneous dual image acquisition. Pictures were taken in an interval of 2 s for EB3 measurements and taken continuously for BDNF vesicle analysis. The whole time interval for each video clip was 5 min, at least 10 videos were performed for each cell line. All experiments were performed at least three times.

### Tracking software

Cell tracking was performed by the software Volocity 6.0 3D Image Analysis Software (PerkinElmer). Tracking was performed manually, velocity and track length were analyzed by using the Volocity software. For analysis of EB3 movements, at least 10 EB3 signals at microtubule plus ends per cell were tracked. For BDNF vesicle trafficking, the number of bidirectionally moving vesicles, vesicle accumulations per cell and number of linearly moving vesicles per cell were counted by eye, track length of vesicles with linear movement was analyzed by manual tracking.

### Statistical analysis

Statistical comparisons of normalized values in two groups were performed using Student's *t*-test for unpaired samples. Values ≤ 0.05 were taken to indicate significant differences between groups.

## SUPPLEMENTARY FIGURES


