# Supplementary material for: Drosophila homologue of Diaphanous 1 (DIAPH1) controls the metastatic potential of colon cancer cells by regulating microtubule-dependent adhesion
Source: Oncotarget. 2015 May 25;6(21):18577–89. doi: 10.18632/oncotarget.4094 (PMC4621911; doi:10.18632/oncotarget.4094)
Supplement: Supplementary file 1 [file oncotarget-06-18577-s001.pdf]

SUPPLEMENTARY FIGURES

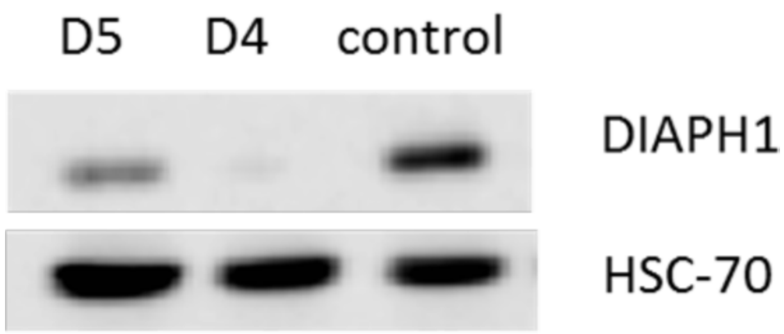

Supplementary Figure S1: With shRNA 4 (D4) an almost 95%, and with shRNA 5 (D5) a 60% down-regulation of DIAPH1 was achieved. Cells stably expressing scrambled shRNA served as control. D4 and D5: DIAPH1 knock down cells, control: scrambled cells, HSC-70: loading control.

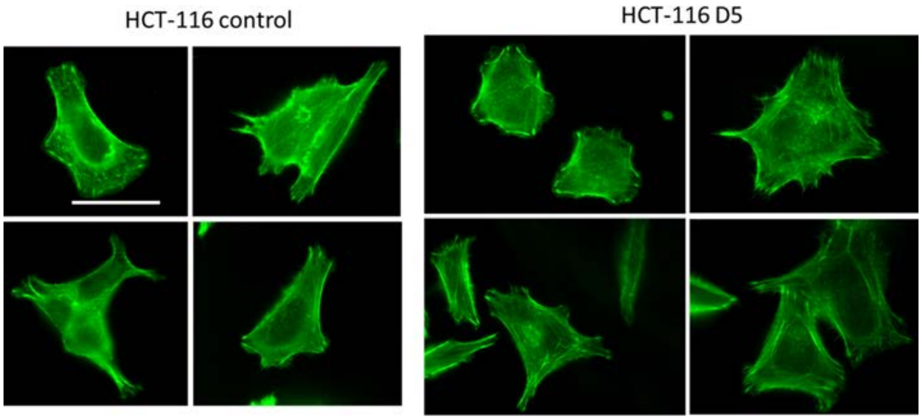

Supplementary Figure S2: Phalloidin-staining of actin filaments in HCT-116 control and DIAPH1-depleted cells. Paraformaldehyde-fixed cells were treated with Alexa-flour488-coupled phalloidin and phalloidin-derived fluorescence was analyzed by fluorescence microscopy. Determination of fluorescence-intensity of at least 100 cells per group did not show a significant difference between control and DIAHP1-depleted cells (D5). Shown are representative micrographs of both cell lines. Bar: 10 µm.
